# Ex vivo RSA and *pfkelch13* targeted-amplicon deep sequencing reveal parasites susceptibility to artemisinin in Senegal, 2017

**DOI:** 10.1186/s12936-023-04588-1

**Published:** 2023-05-26

**Authors:** Mamadou Samb Yade, Baba Dièye, Romain Coppée, Aminata Mbaye, Mamadou Alpha Diallo, Khadim Diongue, Justine Bailly, Atikatou Mama, Awa Fall, Alphonse Birane Thiaw, Ibrahima Mbaye Ndiaye, Tolla Ndiaye, Amy Gaye, Abdoulaye Tine, Younouss Diédhiou, Amadou Mactar Mbaye, Cécile Doderer-Lang, Mamane Nassirou Garba, Amy Kristine Bei, Didier Ménard, Daouda Ndiaye

**Affiliations:** 1 Laboratory of Parasitology-Mycology, Aristide le Dantec Hospital, Université Cheikh Anta Diop, Dakar, Senegal; 2 International Research Training Center on Genomics and Health Surveillance (CIGASS), Dakar, Senegal; 3 Université Paris Cité and Sorbonne Paris Nord, Inserm, IAME, 75018 Paris, France; 4 Université Gamal Abdel Nasser de Conakry/Centre for Research and Training in Infectiology of Guinea (CERFIG), Conakry, Guinea; 5 Service of Parasitology-Mycology, Faculty of Medecine, Pharmacy, and Odontostomatology, Cheikh Anta Diop University of Dakar, Dakar, 10700 Senegal; 6 Université Paris Cité, IRD, MERIT, 75006 Paris, France; 7 Université de Paris, Institut Cochin, Inserm U1016, Service de Parasitologie Hôpital Cochin, 75014 Paris, France; 8 Department of Biochemistry and Functional Genomics, Faculty of Medicine and Health Sciences, Université de Sherbrooke, Sherbrooke, Canada; 9 Université de Strasbourg, Institute of Parasitology and Tropical Diseases, UR7292 Dynamics of Host-Pathogen Interactions, 67000 Strasbourg, France; 10 Department of Epidemiology of Microbial Diseases, Yale School of Public Health, New Haven, CT USA; 11 Department of Immunology and Infectious Diseases, Harvard TH Chan School of Public Health, Boston, MA 02115 USA; 12 CHU Strasbourg, Laboratory of Parasitology and Medical Mycology, 67000 Strasbourg, France; 13 Institut Pasteur, Université Paris Cité, Malaria Genetics and Resistance Unit, INSERM U1201, 75015 Paris, France; 14 Institut Pasteur, Université de Paris, Malaria Parasite Biology and Vaccines Unit, Paris, France

**Keywords:** Malaria, *Plasmodium falciparum*, Artemisinin partial resistance, Ring-stage Survival Assay, *Pfkelch13* genotype, Targeted-amplicon deep sequencing, Senegal

## Abstract

**Background:**

Malaria control is highly dependent on the effectiveness of artemisinin-based combination therapy (ACT), the current frontline malaria curative treatment. Unfortunately, the emergence and spread of parasites resistant to artemisinin (ART) derivatives in Southeast Asia and South America, and more recently in Rwanda and Uganda (East Africa), compromise their long-term use in sub-Saharan Africa, where most malaria deaths occur.

**Methods:**

Here, ex vivo susceptibility to dihydroartemisinin (DHA) was evaluated from 38 *Plasmodium falciparum* isolates collected in 2017 in Thiès (Senegal) expressed in the Ring-stage Survival Assay (RSA). Both major and minor variants were explored in the three conserved-encoding domains of the *pfkelch13* gene, the main determinant of ART resistance using a targeted-amplicon deep sequencing (TADS) approach.

**Results:**

All samples tested in the ex vivo RSA were found to be susceptible to DHA (parasite survival rate < 1%). The non-synonymous mutations K189T and K248R in *pfkelch13* were observed each in one isolate, as major (99%) or minor (5%) variants, respectively.

**Conclusion:**

The results suggest that ART is still fully effective in the Thiès region of Senegal in 2017. Investigations combining ex vivo RSA and TADS are a useful approach for monitoring ART resistance in Africa.

## Background

Prompt management of malaria cases remains a vital component of malaria control and elimination strategies [1]. Over the two last decades, artemisinin (ART)-based combination therapy (ACT) has contributed significantly to the reduction of malaria-related morbidity and mortality in sub-Saharan Africa [2]. Since 2006, the Senegalese National Malaria Control Programme (NMCP) has recommended artemether–lumefantrine (AL) as the first-line treatment for uncomplicated malaria [3]. However, recent reports of the emergence and expansion of partial resistance to ART (ART-R) in the Greater Mekong Subregion have endangered the long-term efficacy of ACT. Although ACT remains clinically and parasitologically effective in most African malaria endemic countries [4], the local emergences of ART-R in Rwanda and in Uganda, respectively reported in 2020 and 2021, are warning signals to the loss of ART efficiency [5–8]. As ART-R could independently emerge in western Africa or spread from eastern African ATR-R hotspots, the monitoring of *Plasmodium falciparum* parasites susceptibility to ART using the current clinical and biological tools is of utmost importance and must be implemented [4]. Clinically, ART-R is defined as delayed parasite clearance or Day-3 positive parasitaemia upon ART-based treatment [9]. Delayed clearance has been associated with decreased in vitro parasite susceptibility (survival rate ≥ 1%) expressed in the Ring-stage Survival Assay (RSA^0–3 h^) [10]. These clinical and biological phenotypes were later associated with non-synonymous mutations in the *P. falciparum kelch13* gene (*pfkelch13*) [11]. *Pfkelch13* encodes a 726-amino acid protein containing three structurally conserved domains: a coiled-coil-containing (CCC) domain, a Broad-Complex, Tramtrack and Bric a brac (BTB) domain, and a C-terminal kelch-repeat propeller domain where most of non-synonymous mutations associated with ART-R have been described [12]. Since then, a surveillance based on the detection of *pfkelch13* single-nucleotide polymorphisms (SNPs) has been conducted in many malaria endemic settings. In the Greater Mekong Subregion, multiple ART-R *pfkelch13* mutant parasites evolved concomitantly until a multidrug-resistant *pfkelch13* C580Y lineage (named *KEL1/PLA1*) outcompeted the others and spread across Southeast Asia [13]. In Africa, this variant has been sporadically reported [14–16]. Rather, the *pfkelch13* R561H mutant rapidly increased in frequency in Rwanda between 2014 and 2016 and 2018–2019 (from 8 to 22%, respectively) [17–19] and was associated with in vivo and in vitro ART-R [19]. Similarly, in Uganda, two *pfkelch13* mutants (A675V and C469Y) were recently reported to be associated with in vivo and in vitro ART-R [6, 8, 20]. In Senegal, most investigations have looked for SNPs in the propeller-encoding domain of the *pfkelch13* gene using conventional methods, such as PCR/Sanger sequencing [21–26], except for two studies that used *pfkelch13* targeted-amplicon deep sequencing (TADS) [25, 26]. Although the authors did not detect validated or candidate ART-R *pfkelch13* mutations [27], the TADS approach is particularly relevant to detect the presence of minor variants in *P. falciparum* isolates. Here, the *pfkelch13* genotype was evaluated by TADS in 38 *P. falciparum* isolates collected in Thiès in 2017. The genetic variation was explored in the three conserved-encoding domains of *pfkelch13* owing that one mutation in the BTB-encoding domain of *pfkelch13* gene in a western African strain has been shown to confer ART-R [28].

## Methods

### Ethics

The National Ethics Committee for Health Research and the Ministry of Health of Senegal approved the protocol used for this study under number 00000169/MSAS/DPRS/CNERS (December 2, 2016). Written and informed consent was obtained from all participants, before participant recruitment and sample collection.

### Study site

The study was conducted during the peak malaria season (September to December) in 2017 in Thiès, a city ~ 75 km southeast of Dakar (Senegal; 14° 47′ 26″ north, 16° 55′ 29″ west), an area belonging to the Sahelian facies defined by a short malaria seasonal transmission (< 4 months). In this region, the entomological inoculation rate (EIR) is low and varies from one year to another (0–20 infectious bites/person/year), estimated to be < 5 [29], and malaria is mainly transmitted by *Anopheles arabiensis* and *Anopheles gambiae* mosquito vectors. Malaria transmission is perennial and hypoendemic, and malaria incidence ranges between 5 and 15 per 1000 inhabitants [30].

### Study design

Individuals who visited clinic of the Service de Lutte Antipaludique (SLAP) harbouring a *P. falciparum* infection only, confirmed by thick drop and thin smear; a parasite density between 1000 and 200,000 asexual forms/µL of blood; an absence of general danger signs or other signs of severe and complicated *P. falciparum* malaria as defined by the World Health Organization (WHO) [31]; a fever or history of fever within 48 h; absence of history of hypersensitivity reactions to the drugs assessed; living within 10 km of the health facility; and a provided informed consent (adult and/or equivalent minor), or legal guardian consent in the case of children, were enrolled. Patients with clinical malaria were treated with artemether–lumefantrine (AL, Coartem®), according to the treatment guideline of the Senegal National Malaria Control Programme (NMCP). For each patient, 5 mL vacutainer tubes of venous blood were collected for ex vivo RSA.

### Parasite culture and ring-stage survival assay

Parasitaemia was estimated by microscopy examination on Giemsa-stained blood smears. Venous blood samples were then processed by eliminating plasma, leukocytes and anticoagulant from red blood cells (RBCs), washed twice in RPMI 1640 medium (Gibco, Life technologies). Parasitaemia were adjusted to 1% if greater by adding uninfected RBCs as previously described [10]. Then, 900 µL RBCs were loaded into wells, exposed to either 100 µL of 700 nM DHA (Sigma, reference D7439) or 0.1% of dimethyl sulfoxide (DMSO; Sigma, reference D8418) and cultivated at 37 °C in incubator for 6 h (conditions: 94% N_2_, 5% CO_2_, 1% O_2_). Finally, RBCs were washed and cultivated for 66 h. The proportions of viable parasites were estimated independently by two expert malaria microscopists on Giemsa-stained thin smears. The number of viable parasites that developed into ring/trophozoite stages were determined, pyknotic forms were excluded [32]. The average of the two counts was calculated. If any discrepancy was noted (either difference of parasite density of > 50%), slides were checked by a third independent reader, and parasite densities were calculated by averaging the two most close counts. Survival rates were calculated as the ratio of parasites in exposed and non-exposed cultures. Results were deemed as interpretable if the parasitaemia in the sample exposed to DMSO was higher than the initial parasitaemia at 0 h [33]. The *P. falciparum* ART-sensitive 3D7 strain was used as a negative control.

### DNA extraction

DNA was extracted from the same whole blood sample used for the ex vivo RSA using the QIAamp DNA Blood Mini Kit (Qiagen, Valencia, CA, USA) according to manufacturer’s instructions.

### Targeted-amplicon deep sequencing of *pfkelch13*

Extracted DNA samples were subjected to two separate overlapping PCRs covering the three conserved-encoding domains of the *pfkelch13* gene (Jérôme Clain, personal communication). The first fragment covered both the CCC and BTB domains (5′-agatgcagcaaatctta-3′ and 5′-ttctacaccatcaaatcc-3) and measured 850 bp; and the second fragment covered from the end of the CCC domain to the end of the propeller domain (5′-aaaaagaaaaagaagaacataggaaa-3′ and 5′-tgtgcatgaaaataaatattaaagaag-3′) and measured 1,409 bp. Briefly, 1 µL of DNA was amplified with 0.25 µM of each primer, 0.2 mM of dNTP, 0.625 unit of GoTaq G2 Flexi DNA Polymerase (Promega, Madison, USA) and 2.5 mM and 3 mM of MgCl_2_ for the first and the second fragment, respectively. The cycling conditions were as follows: 3 min at 95 °C, then 40 cycles of 30 s at 95 °C, 30 s at 55 °C, 90 s at 68 °C and final extension 3 min at 68 °C. PCR products were detected using 1% agarose gel electrophoresis and SYBR Safe staining (Invitrogen, Waltham, USA). DNA from *P. falciparum* 3D7 strain and water were used as positive and negative controls, respectively.

Generated amplicons were then sequenced by next-generation sequencing. Briefly, PCR amplicons were fragmented with the Covaris S220 to about 150 bp, and libraries were constructed using the KAPA HyperPrep Library Preparation Kit (Kapa Biosystems, Woburn, MA) following the manufacturer’s protocol. Amplicons were purified with AMPure Agencourt XP beads (Beckman Coulter). Libraries quality and quantity control were assessed using Qubit® for concentration and Bio Analyser 2100 Agilent for fragment size. Libraries were pooled at approximately equal concentration and sequenced on an Illumina NextSeq 500 instrument (Illumina Inc, San Diego, CA, USA) to generate 150 bp paired-end reads at the GENOM’IC platform of Cochin Institute (Paris, France). Raw sequences were then demultiplexed and quality trimmed at a Phred score of 30. Primer sequences were trimmed from the 5′-end of the sequences to avoid primer bias in the sequenced fragments. Base calling was performed by comparing reads with a custom database consisting of the *pfkelch13* sequence retrieved from the 3D7 reference genome. Bioinformatic analyses were performed using the CLC Genomics Workbench 22 software (Qiagen), using the following criteria: an allele was studied when its frequency was > 2%; the allele was considered as minor when the frequency was < 50%; otherwise, the allele was considered as major.

## Results

### Baseline characteristics

A total of 38 patients with uncomplicated *P. falciparum* malaria meeting the inclusion criteria were enrolled. The sex ratio (M/F) was largely dominated by males (36 males/2 females). The age of the participants ranged from 9 to 70 years (median of 21.5 years). Median weight was 59.5 kg and median body temperature was 38.5 °C. The median parasitaemia was 1.03%, ranging from 0.68 to 1.53% (Table 1).

**Table 1:** Baseline characteristics of the study participants and the *P. falciparum* isolates, Thiès, Senegal, 2017

	Median	CI_95%_	IQR_25 − 75_
Age (years)	21.5	17.0–25.0	15.0–28.0
Weight (kg)	59.5	50.5–64.5	38.0–67.0
Temperature (°C)	38.5	38.2–39.1	37.8–39.5
Glycaemia	0.9	0.9–10.9	0.8–1.0
Haemoglobin	13.4	12.1–14.2	11.5–15.4
Parasitaemia (%)	1.0	0.7–1.5	0.6–1.9

CI_95%_: 95% confidence interval; IQR_25-75_: Interquartile range

### Ex vivo RSA phenotype

Examination of blood smears and species-specific PCR revealed that all samples were *P. falciparum* monoinfections. Ex vivo RSA were successfully performed for the 38 tested samples. Survival rates showed the absence of surviving parasite to the 700 nM DHA pulse as for the 3D7 strain (0%) except for three isolates Th54, Th77 and Th95 (0.054%, 0.06% and 0.22% respectively) which were however less than the 1% threshold to be considered as associated with ART-R.

### *Pfkelch13* genotyping

Among the 38 samples successfully sequenced, two non-synonymous mutations (K189T and K248R) located outside of the propeller-encoding domain of *pfkelch13* were detected (Table 2). Each mutation was observed in a single sample in major (99% for K189T) and minor (5% for K248R) proportions. The *pfkelch13* K248R mutant was detected for the first time in Senegal (Table 3).

**Table 2:** Proportion of non-synonymous *pfkelch13* mutations detected in the 38 *P. falciparum* isolates

Codon position	Reference sequence		Mutant sequence		Type	n/N
	Amino acid	Nucleotide	Amino acid	Nucleotide		
189	K	AAA	T	A**C**A	NS	1/38
248	K	AAG	R	A**G**G	NS	1/38

The boldface highlights the nucleotide base change. n: number of samples harbouring the mutant allele; N: total number of successfully sequenced samples; NS: non-synonymous mutation

**Table 3:** List of the mutations in the *pfkelch13* gene previously observed in Senegal and in the present study

*Plasmodium*-specific/CCC/BTB-POZ	Propeller	Authors	Year(s)	City
T149S and K189T* N142N/NN (insertion)	No mutation	Madamet et al.	2012–2013	Dakar
D109H, L119L, H136N, P152P, M163I, K189T*, K189N, D214G, M235I, R239L, R255K, L258M, K278N, G287C, K293N, I313I, G357V, T367T, L368I, R398M, S423I, L429F	P443Q, S459S, Q468Q, C469C, C473F, W518C, G533V, R539I, R553I, V555L, E556V, P570L, A578D, R587I, G592V, R597I, A621A, A626V, W660C, G690G	Talundzic et al.	2011–2014	Dakar and Thiès
K123R, N137S, N142NN/NNN, T149S, and K189T/N	N554H, Q613H, and V637I	Boussaroque et al.	2013–2014	Dakar
D109H, T149S, K189N, K189T*, H274Y, D283Y, D389Y, N141-N142NN, N142N	T478T, A578S and V637I	Gaye et al.	2015–2016	Dakar (2015), Kédougou and Matam (2016)
No mutation	C469C, F491F, G545G, F583F and A627A	Ahouidi et al.	2015, 2016 and 2017	Bounkiling
No mutation	No mutation	Diallo et al.	2018	Diourbel and Kédougou
No mutation	No mutation	Delandre et al.	2015, 2016, 2017, 2018 and 2019	Dakar
K189T*, K248R	No mutation	This Study	2017	Thiès

*Most frequent mutations

## Discussion

ACT is the mainstay of treatment for uncomplicated malaria in malaria endemic regions [2]. Unfortunately, the emergence and the clonal expansion of *pfkelch13* mutant parasites have been reported recently in Rwanda (R561H) and Uganda (C469Y and A675V) [5–8]. Therefore, the WHO recommends to closely monitor the susceptibility of *P. falciparum* to anti-malarial drugs and particularly to ART derivatives [4]. As in many sub-Saharan African countries, ACT has contributed significantly to the decline in malaria incidence and mortality over the past decade. In Senegal, considerable efforts have been done to reduce malaria morbidity and mortality mainly since 2006 when AL was introduced [3]. Consequently, the emergence of ART-resistant parasites is a major threat which can hinder malaria elimination.

Partial resistance to ART is associated with non-synonymous mutations in the *pfkelch13* gene. However, the impact of most mutations on ART-R detected in field samples is largely unknown mainly due to the lack of association between the clinical phenotype (delayed parasite clearance) and the *pfkelch13* genotype. The study presented here aims to fill this gap by combining ex vivo RSA with *pfkelch13* genotyping. The ex vivo RSA estimates the susceptibility of *P. falciparum* to ART using parasite isolates freshly collected from patients with malaria. The ex vivo RSA has been previously used in studies conducted in Central [34] and East Africa [33, 35]. Four isolates from Uganda showed high (> 10%) survival rates, levels of which are reported to be closely associated with delayed parasite clearance following artesunate monotherapy [33]. The data presented here showed that none of the tested samples confer in vitro ART-R with a survival rate ≥ 1%, suggesting the absence of decrease susceptibility of Senegalese parasites to ART derivatives in 2017.

By using a TADS approach, two *pfkelch13* non-synonymous mutations (K189T and K248R) were detected. The K248R mutation was observed in one sample in minor proportion (5%). The mutation is located in the CCC domain of *pfkelch13*, but the mutation is not likely related to ART-R since the survival rate was less than 1%. While K189T had already been reported in Africa [36, 37], the non-synonymous mutation K248R was detected for the first time in Senegal. The isolate carrying the K189T mutation had also a survival rate < 1%, confirming that this allele does not confer in vitro ART-R as previously observed in India [38]. Only three *pfkelch13* wild-type isolates (Th54, Th77 and Th95) were found to have a survival rate above 0% (but still < 1%). To date, in vitro susceptibility to ART derivatives by RSA of *P. falciparum* isolates have been reported in Cameroon and Uganda. Two studies conducted in Cameroon [34, 35] showed the absence of *pfkelch13* mutations associated with ART-R while one study in Uganda [33] reported high survival rates of isolates carrying the *pfkelch13* A675V and C469Y mutations.

The study presented here has several limitations. First, this work included only 38 samples. Second, despite this low sample size, systematic ex vivo RSA is time consuming: parasites exposure to DHA is 6 h before washing the drug, several samples cannot be processed at the same time and the method requires skilled personnel, and all validation steps must be done by microscopy experts. Third, in vitro RSA (from culture-adapted parasites) was not performed to confirm the ex vivo survival rates. And fourth, the study was conducted only in one site in Senegal (Thiès) and no clinical data was collected.

## Conclusion

This study shows that combining ex vivo RSA phenotype and *pfkelch13* genotyping can be efficiently carried out to monitor ART-R, providing relevant data to malaria control programs on parasite susceptibility to ART. Particularly, TADS used here confirmed to be useful to detect the presence of minor alleles. This study showed that all *P. falciparum* isolates collected in Thiès (Senegal) in 2017 were susceptible to DHA. As recommended by the WHO [4], similar studies must be conducted in Senegal and other African countries to strengthen surveillance of anti-malarial drug efficacy and ART-R.

## Data Availability

The data that support the findings of this study are available from the corresponding author on reasonable request. All relevant data are within the manuscript.

## Abbreviations

ACT: Artemisinin-based combination therapy
RSA: Ring-stage Survival Assay
TADS: Targeted-amplicon deep sequencing
PCR: Polymerase chain reaction
DNA: Deoxyribonucleic acid
DHA: Dihydroartemisinin
AL: Artemether-lumefantrine
ART: Artemisinin
ART-R: Artemisinin resistance
CCC: Coiled-coil-containing
BTB: Broad-Complex, Tramtrack and Bric a brac
SNP: Single nucleotide polymorphism
RBC: Red blood cell
NMCP: National Malaria Control Programme
WHO: World Health Organization
